# Prevalence of meropenem-resistant *Pseudomonas Aeruginosa *in Ethiopia: a systematic review and meta‑analysis

**DOI:** 10.1186/s13756-024-01389-2

**Published:** 2024-04-10

**Authors:** Mengistie Yirsaw Gobezie, Minimize Hassen, Nuhamin Alemayehu Tesfaye, Tewodros Solomon, Mulat Belete Demessie, Tesfaye Dessale Kassa, Teklehaimanot Fentie Wendie, Abel Andualem, Ermiyas Alemayehu, Yaschilal Muche Belayneh

**Affiliations:** 1 Department of Clinical Pharmacy, School of Pharmacy, College of Medicine and Health Sciences, Wollo University, Dessie, Ethiopia; 2 Department of Pharmacology, School of Pharmacy, College of Medicine and Health Sciences, Wollo University, Dessie, Ethiopia; 3 Department of Clinical Pharmacy, College of Health Sciences, Mekelle University, Mekelle, Ethiopia; 4 Department of Medical Laboratory Sciences, College of Medicine and Health Sciences, Wollo University, Dessie, Ethiopia

**Keywords:** Meropenem Resistant, Pseudomonas Aeruginosa, Systematic review, Meta-analysis, Ethiopia

## Abstract

**Introduction:**

Antimicrobial resistance (AMR) is a pressing global health concern, particularly pronounced in low-resource settings. In Ethiopia, the escalating prevalence of carbapenem-resistant Pseudomonas aeruginosa (*P. aeruginosa*) poses a substantial threat to public health.

**Methods:**

A comprehensive search of databases, including PubMed, Scopus, Embase, Hinari, and Google Scholar, identified relevant studies. Inclusion criteria encompassed observational studies reporting the prevalence of meropenem-resistant *P. aeruginosa* in Ethiopia. Quality assessment utilized JBI checklists. A random-effects meta-analysis pooled data on study characteristics and prevalence estimates, with subsequent subgroup and sensitivity analyses. Publication bias was assessed graphically and statistically.

**Results:**

Out of 433 studies, nineteen, comprising a total sample of 11,131, met inclusion criteria. The pooled prevalence of meropenem-resistant *P. aeruginosa* was 15% (95% CI: 10–21%). Significant heterogeneity (I^2^ = 83.6%) was observed, with the number of *P. aeruginosa* isolates identified as the primary source of heterogeneity (*p* = 0.127). Subgroup analysis by infection source revealed a higher prevalence in hospital-acquired infections (28%, 95% CI: 10, 46) compared to community settings (6%, 95% CI: 2, 11). Geographic based subgroup analysis indicated the highest prevalence in the Amhara region (23%, 95% CI: 8, 38), followed by Addis Ababa (21%, 95% CI: 11, 32), and lower prevalence in the Oromia region (7%, 95% CI: 4, 19). Wound samples exhibited the highest resistance (25%, 95% CI: 25, 78), while sputum samples showed the lowest prevalence. Publication bias, identified through funnel plot examination and Egger’s regression test (*p* < 0.001), execution of trim and fill analysis resulted in an adjusted pooled prevalence of (3.7%, 95% CI: 2.3, 9.6).

**Conclusion:**

The noteworthy prevalence of meropenem resistance among *P. aeruginosa* isolates in Ethiopia, particularly in healthcare settings, underscores the urgency of implementing strict infection control practices and antibiotic stewardship. Further research is imperative to address and mitigate the challenges posed by antimicrobial resistance in the country.

## Introduction

Antimicrobial resistance (AMR) is a leading cause of death around the world, with the highest burdens in low-resource settings [1]. Antimicrobial resistance can be stated as the innate or acquired capacity of a microbe to impede the effectiveness of an antimicrobial medication to the point where it is no longer effective. Antibiotic susceptibility test is the gold standard for diagnosing bacterial resistance and directing clinicians in the proper and prompt management of bacterial infections and is mostly conducted with a disk diffusion test method [2].

*P. aeruginosa* is the most commonly encountered human pathogen in the family pseudomonadaceae which is characterized by a gram-negative, straight or slightly curved rod with a length ranging from 1 to 3 μm and a width of 0.5 to 1.0 μm [3, 4].

*P. aeruginosa* most commonly infects individuals with severe burns, tuberculosis, cancer, and AIDS. It also causes infections in the urinary tract, respiratory system, dermis, soft tissue, bacteraemia, bone and joint, gastrointestine, and blood. Significantly, *P. aeruginosa* poses a 50% risk to patients hospitalized with burns, cystic fibrosis, and cancer [5].

The prevalence of *P. aeruginosa* has been increased dramatically. The center for disease control and prevention have been proclaimed that *P. aeruginosa* infection is the fourth most common isolated nosocomial pathogen in US which accounts about 10% of all hospital-acquired infections [6]. Reports from Iran also confirm that it is among the most common causes of nosocomial infections [7]. Not only in the developed countries, has the prevalence of *P. aeruginosa* also risen in developing countries. The pooled prevalence of *P. aeruginosa* in Africa rises to 11.8% in a recent report [8] and it may be inclined beyond these level because of the low awareness of antimicrobial resistance, the empirical therapy treatment approach due to the lack of appropriate microbial susceptibility test and the improper use of antibiotic in the continent.

Currently most of the pseudomonas infections are treated with carbapenems. Meropenem is a broad-spectrum carbapenem antibiotic which is effective against both Gram-positive and Gram-negative bacteria. It attains its action by easily getting into bacterial cells and inhibiting the production of essential cell wall components, which results in cell death [9].

The three extensively researched chromosomally encoded resistance mechanisms against carbapenems in Pseudomonas aeruginosa include: deactivation of the outer membrane protein OprD; heightened expression of chromosome-encoded ampC (β–lactamase); and increased production of multidrug efflux pumps like MexAB–OprM and MexXY–OprM [10, 11].

The utilization of carbapenems and the emergence carbapenem resistant *P. aeruginosa* is increased concomitantly in dramatic way throughout the different region of the world [12, 13]. A significant increase in resistance of carbapenem to the gram negative bacilli including the *P. aeruginosa* was recorded which ranges from 30.1 to 74% [14–18]. To reduce the antibiotic resistance of such nosocomial infections, these must be investigated globally and interventions must be implemented. Considering of this, the purpose of this systematic review and meta-analysis was to assess the prevalence of meropenem resistant *P. aeruginosa* and the resistance.

## Methods

### Reporting

The results of this review were reported in accordance with the Preferred Reporting Items for Systematic Reviews and Meta-analysis (PRISMA) guideline [19]. The protocol for this systematic review and meta-analysis has been registered in the Prospero database under the registration number PROSPERO 2023: CRD42023441292.

### Databases and search strategy

A comprehensive search was conducted across multiple electronic databases, including PubMed, Google Scholar, Hinari, SCOPUS, and EMBASE. The search strategy, devised by three authors, was executed by another three authors from June 20–30, 2023. The search terms employed were: “prevalence” AND “meropenem resistant *Pseudomonas aeruginosa* OR meropenem resistant *p. aeruginosa* OR carbapenem resistant *Pseudomonas aeruginosa* OR multidrug-resistant *Pseudomonas aeruginosa* OR meropenem resistant gram-negative bacteria OR meropenem resistant Enterobacteriaceae” AND “Ethiopia”. The Boolean operators “OR” and “AND” were used as appropriate. Additionally, the proceedings of professional associations and university repositories were scrutinized. A direct Google search was conducted, and bibliographies of identified studies were reviewed to include any relevant studies inadvertently omitted during electronic database searches. The PubMed search query is as follows: (((((((“meropenem resistan*“[Title/Abstract]) OR (“carbapenem resistan*“[Title/Abstract])) AND (“Pseudomonas aeruginosa“[Title/Abstract])) OR (p.aeruginosa[Title/Abstract])) OR (“multidrug-resistant Pseudomonas aeruginosa“[Title/Abstract])) OR (Enterobacteriaceae[Title/Abstract])) OR (“gram-negative bacteria“[Title/Abstract])) AND (Ethiopia[Title/Abstract]).The management of references and removal of duplicates were handled using Endnote 20 software [20].

### Inclusion and exclusion criteria

The search encompassed studies published prior to the search date. Studies were considered eligible if they met the following inclusion criteria: [1] focused on the prevalence of meropenem-resistant *P. aeruginosa*; [2] designed as observational studies, including cross-sectional studies, randomized controlled trials, or surveillance studies; [4] conducted in Ethiopia; and [5] published in the English language. Exclusion criteria comprised case reports, case-control studies, reviews, commentaries, editorials, and conference abstracts, which were not actively sought during the search process.

### Screening and quality assessment

To eliminate duplicate studies, we utilized Endnote version 20 [20] as our reference manager. Two authors (MY and TD) independently scrutinized the titles and abstracts to identify articles for further consideration in the full-text review. The full text of the remaining articles was then obtained, and two investigators, NA and MH, independently conducted eligibility assessments and subsequently evaluated the quality of the studies using the JBI critical appraisal checklist designed for studies reporting prevalence data [21].

The JBI critical appraisal checklist encompassed various criteria, including [1] the appropriateness of the sampling frame to address the target population; [2] the suitability of the study participant sampling technique and the adequacy of the sample size; [3] a detailed description of study subjects and setting; [4] a thorough analysis of the data and the validity and reliability of methods used for measuring the prevalence of meropenem-resistant *P. aeruginosa*; and [5] the appropriateness of statistical analyses and the adequacy of the sample size. Discrepancies were resolved through consensus. Studies scoring five or above out of a total of nine criteria were categorized as low-risk in terms of methodological quality.

### Data extraction

Data extraction was carried out by three authors (MY, NA, and MH) following a predefined data extraction format. In instances of discrepancies, a repeated procedure was employed to ensure accuracy and consistency. The consolidation and summarization of the final set of articles that met our inclusion criteria were performed by TD, TS, and MB. These authors compiled comprehensive tables containing information on authors, study period, publication year, study design, setting, region, sample size, source of infection, sample type, number of total and resistant isolates of *P. aeruginosa*, and the method employed for antimicrobial sensitivity testing.

### Outcome of interest

The primary outcome of interest was to determine the pooled prevalence of meropenem-resistant *P. aeruginosa* in Ethiopia. This was computed by dividing the number of resistant isolates by the total count of *P. aeruginosa* isolates.

### Data analysis

To estimate the prevalence of meropenem-resistant *P. aeruginosa* in Ethiopia, we employed a weighted inverse variance random-effects model [22]. Addressing variations in the pooled prevalence estimates, we conducted subgroup analyses based on the region where the studies were conducted, the source of infection, and the types of samples analyzed. Heterogeneity among the studies was thoroughly examined using a forest plot, meta-regression, and the I^2^ statistic, with 25%, 50%, and 75% denoting low, moderate, and high heterogeneity, respectively [23]. A significance level for the Q test with a *p*-value less than 0.05 was used as an indicator of heterogeneity.

The presentation of results was facilitated through a comprehensive forest plot. To evaluate the potential presence of publication bias, a Funnel plot and Egger’s regression test were employed, where a *p*-value less than 0.05 in Egger’s test suggested significant publication bias. Additionally, Trim and fill analysis were conducted as a supplementary measure to assess publication bias [24].

Ensuring the stability of the summary estimate, a sensitivity analysis was conducted by systematically omitting individual studies. This analysis aimed to gauge the impact of each study on the overall estimate, providing insights into the robustness of the meta-analysis.

The entire meta-analysis was conducted using STATA version 17 [25] a widely recognized statistical software, to ensure precision and reliability in the analysis.

## Result

### Characteristics of included studies

**Fig. 1:**
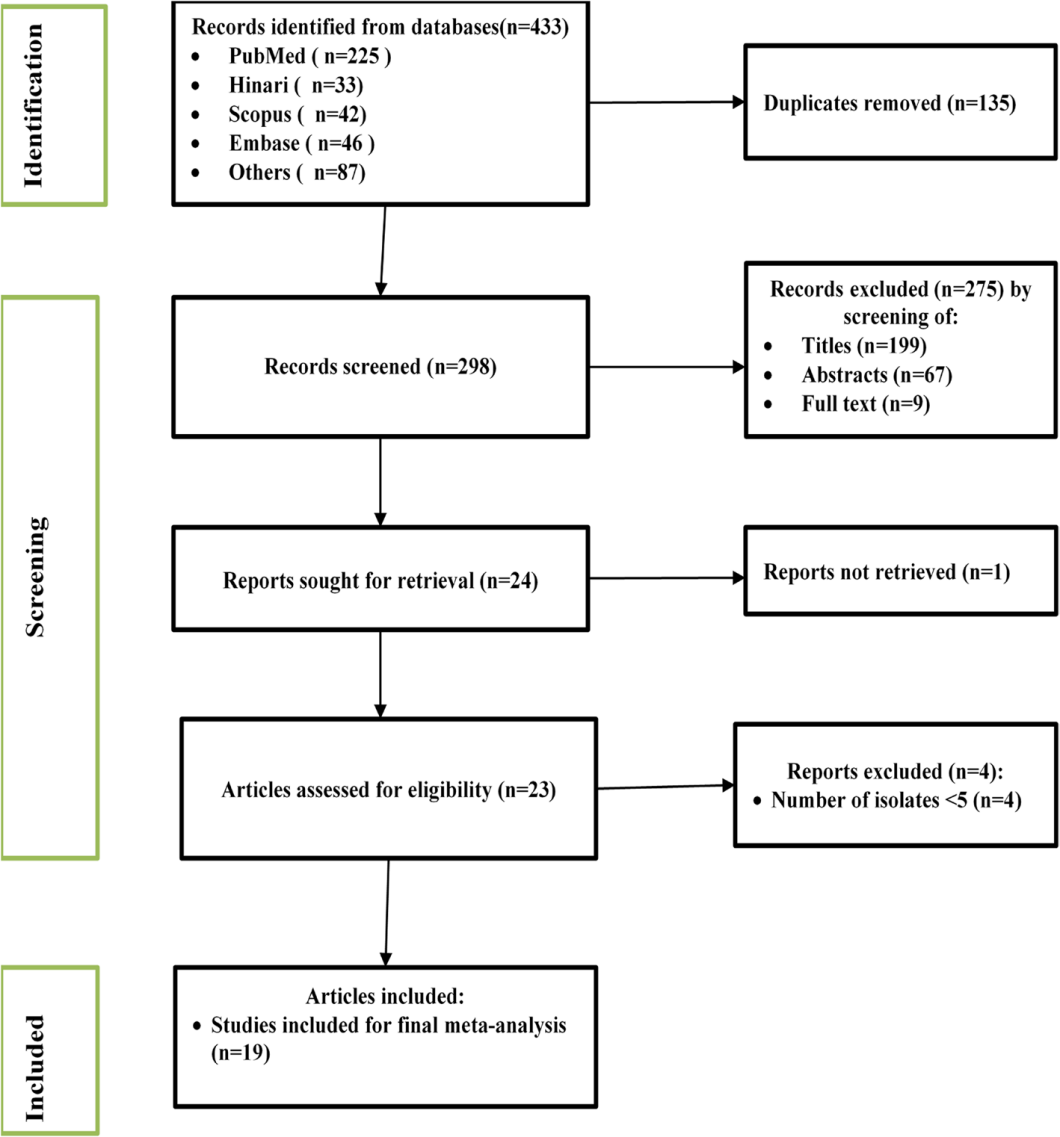
Flow diagram of the included studies for the systematic review and meta-analysis of the prevalence of meropenem resistant P. aeruginosa in Ethiopia

A total of 433 potential studies were identified through various sources, including 225 articles from PubMed, 33 articles from Hinari (research4life), 46 articles from Embase, 42 articles from Scopus, and 87 articles from other sources. The outcomes of the search, along with the reasons for exclusion during the study selection process, are illustrated in Fig. 1.

After thorough scrutiny, 19 articles were deemed suitable for inclusion in the meta-analysis, focusing on assessing the prevalence of meropenem-resistant *P. aeruginosa* in Ethiopia. All included studies adhered to either cross-sectional or cohort study designs. Among these, eight studies specifically investigated the prevalence of meropenem-resistant *P. aeruginosa* in hospital-acquired infections, while the remaining studies were analyzed on community-acquired infections.

Geographically, the studies were distributed across various regions of Ethiopia, with eight conducted in the Amhara region [26–33], three in Oromia [34–36], three in SNNPR [37–39], four in Addis Ababa [40–43], and one in Harari [44].

A comprehensive analysis involved 11,131 study participants, with a focus on 3,109 total isolates identified, out of which 301 were *P. aeruginosa* isolates. For a detailed overview of the included studies, including their characteristics, refer to Table 1.

**Table 1:** Characteristics of studies included for the systematic review and meta-analysis of the meropenem resistant *P. aeruginosa* in Ethiopia

Authors	Year	Region	Total sample	Total No of isolates	No of PA isolate	No of resistant PA	Study Design	Study setting	Study unit	Sample type	Source of infection
Gashaw. et al [34]	2018	Oromia	1015	126	9	4	CS	Hospital	Not defined	Mixed	Hospital
legesse et al [28]	2019	Amhara	1416	426	17	1	CS	Hospitals	Mixed	Mixed	Community
Motbainor. et al [30]	2020	Amhara	238	20	11	5	CS	Hospital	ICU	Mixed	Hospital
Mekonnen et al [29]	2021	Amhara	254	33	18	3	CS	Hospital	ICU	Mixed	Hospital
Alebel et al [27]	2021	Amhara	270	95	11	2	CS	Hospital	ICU	Mixed	Hospital
Abdeta et al [40]	2021	AA	1337	429	36	7	CS	Hospital	Not defined	Mixed	Community
Alemayehu. et al [38]	2021	SNNPR	103	111	8	3	CS	Hospital	Mixed	Mixed	Community
Mitiku et al [39]	2022	SNNPR	422	131	16	4	CS	Hospitals	OPD	Urine	Community
Abda et al [26]	2022	Amhara	153	110	13	2	CS	Hospitals	Not defined	sputum	Community
Bizuayehu et al [43]	2022	AA	220	79	12	2	CS	Hospital	ICU	Urine	Hospital
Abayneh et al [37]	2022	SNNPR	262	41	5	0	Cohort	Hospital	Surgical	wound swabs	Hospital
Worku et al [33]	2022	Amhara	200	54	5	0	CS	Hospital	Oncology	blood	Community
Tilahun et al [32]	2022	Amhara	423	75	46	19	CS	Hospitals	Mixed	Mixed	Hospital
Beshah et al [41]	2022	AA	1490	427	5	2	CS	Hospitals	Mixed	Mixed	Community
Tilahun [31]	2022	Amhara	384	343	31	16	CS	Hospitals	Mixed	wound swabs	Hospital
Sewunet et al [36]	2022	Oromia	1087	80	41	3	CS	Hospitals	Mixed	Mixed	Community
Mussema et al [35]	2022	Oromia	39	32	7	0	CS	Hospitals	Mixed	sputum	Community
Mekonnen. et al [44]	2023	Harari	332	80	5	0	CS	Hospital	Mixed	Mixed	Community
Beshah et al [42]	2023	AA	1486	417	5	2	CS	Hospital	Not Defined	blood	Community

*PA *Pseudomonas aeruginosa*, CS *cross sectional*, SNNPR *southern nation nationalities and people region*, AA *Addis Ababa*, DDM *disk diffusion method*, *Mixed (blood, urine sputum and wound swabs)

### Quality of the included studies

Every study underwent evaluation using the JBI critical appraisal checklist designed for studies reporting prevalence data. The application of JBI quality appraisal checklists revealed that none of the included studies were deemed of poor quality, and as a result, none were excluded from the meta-analysis.

### Meta-analysis

#### Prevalence of Meropenem Resistant *P. Aeruginosa* in Ethiopia

**Fig. 2:**
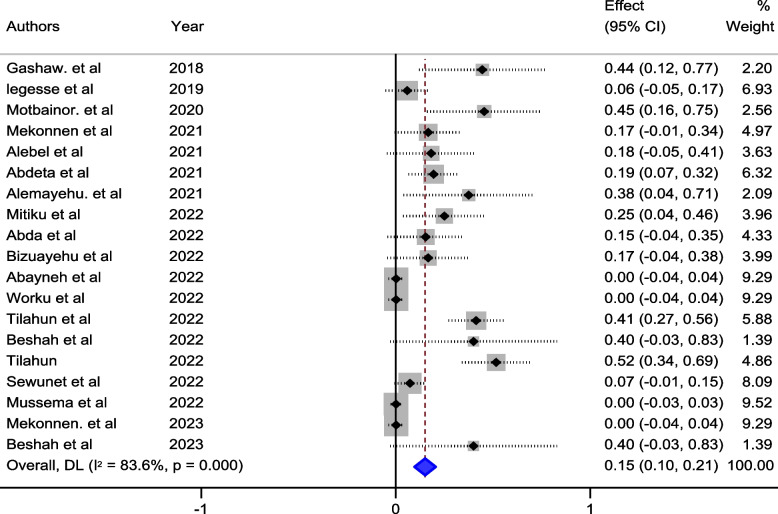
Pooled estimate of prevalence of meropenem resistant *P. aeruginosa* in Ethiopia

Our meta-analysis sought to comprehensively assess the prevalence of meropenem-resistant *P. aeruginosa* in Ethiopia, synthesizing data from multiple studies to derive a pooled estimate. The pooled prevalence of meropenem-resistant *P. aeruginosa* in Ethiopia was 15% (95% Confidence Interval (CI) 10–21, I^2^ = 83.6%; *p* value < 0.001) (Fig. 2).

### Subgroup analysis

**Fig. 3:**
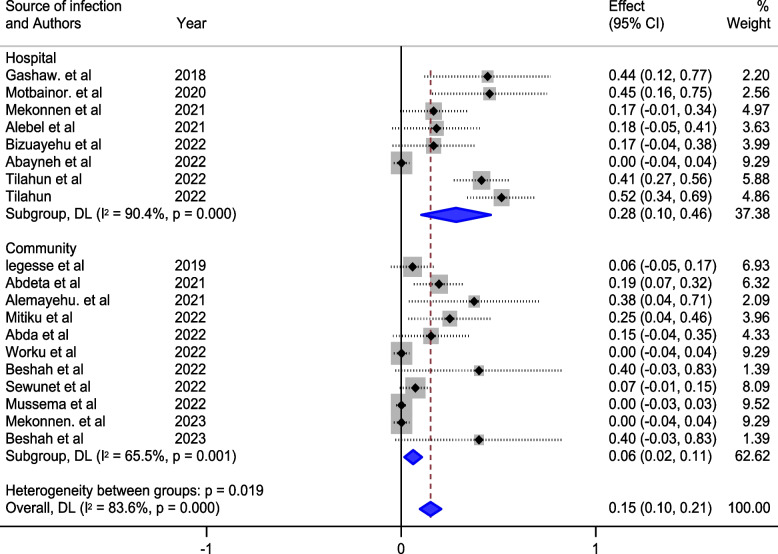
Subgroup analysis of the prevalence of meropenem-resistant *P. aeruginosa* by source of infection

In our detailed subgroup analysis, we delved into the nuanced variations in the prevalence of meropenem-resistant *P. aeruginosa* by categorizing the data based on distinct factors. Firstly, the analysis stratified by the source of infection yielded insightful results. Hospital-acquired infections exhibited a substantial prevalence of 28% (95% CI 10, 46), as depicted in Fig. 3, indicating a notable burden within healthcare settings. In contrast, community-acquired infections revealed a comparatively lower prevalence of 6% (95% CI 2, 11), underscoring distinctions in resistance patterns between hospital and community environments.

**Fig. 4:**
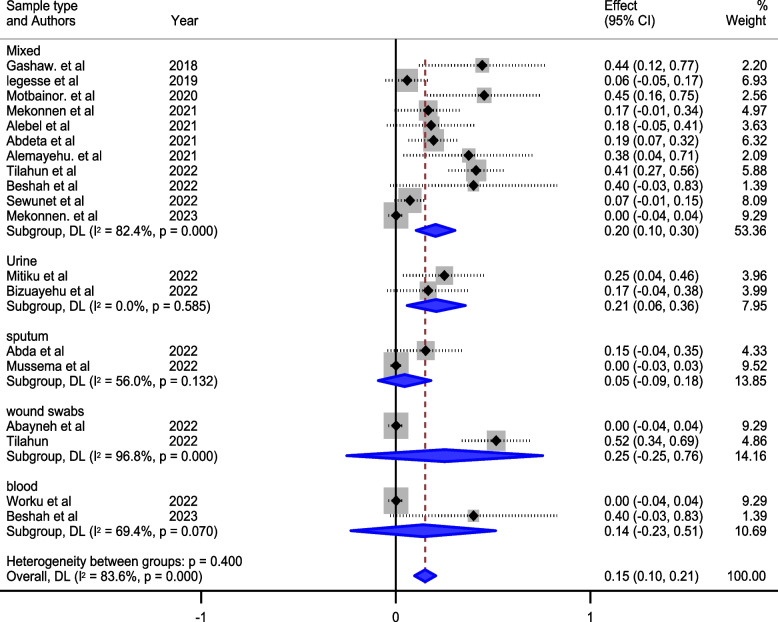
Subgroup analysis of the prevalence of meropenem-resistant *P. aeruginosa* by sample type

Furthermore, our exploration extended to subgroup analysis based on geographical regions and the types of samples tested. Notably, the Examining sample types added another layer of granularity to our findings. Wound swabs, as a specific sample type, exhibited a significant prevalence of 25% (95% CI: 25, 76), as depicted in Fig. 4. Conversely, our analysis revealed a lower prevalence in the Oromia region, standing at 7% (95% CI: 4, 19), and in sputum samples, where the prevalence was 5% (95% CI: 9, 18).

### Heterogeneity analysis

**Fig. 5:**
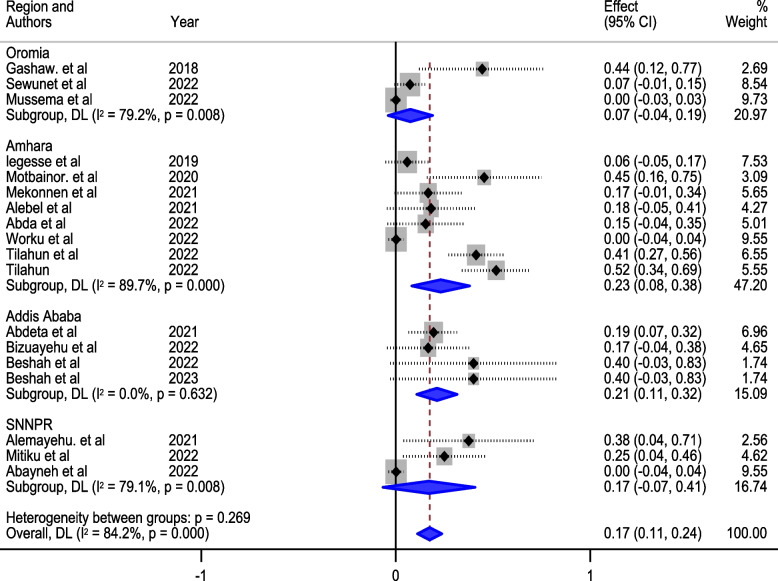
Subgroup analysis of the prevalence of meropenem-resistant *P. aeruginosa* by region

**Fig. 6:**
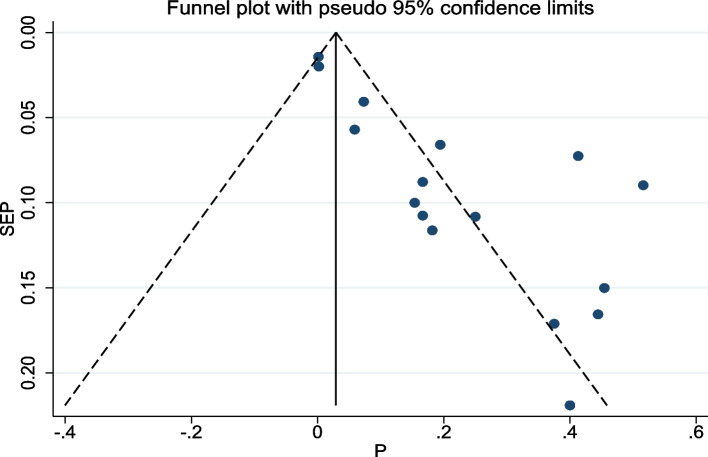
Funnel plot of prevalence of meropenem-resistant *P. aeruginosa* in Ethiopia

The studies incorporated into the analysis exhibited substantial heterogeneity (I^2^ = 83.6%; *p* value < 0.001), and the application of a weighted inverse variance random-effects model did not adequately address this variability. To further explore and understand the heterogeneity, we employed a forest plot (Fig. 2) for subjective assessment and conducted subgroup analyses (Figs. 3, 4 and 5), along with univariate meta-regression utilizing sample size and publication years as variables (Table 2; Fig. 6).

**Table 2:** Meta regression of prevalence of meropenem resistant *P. aeruginosa* and number of isolates and year of study

Variables	Coefficient	Std. Err.	t	P>|t|	[95% Conf. Interval]
**Sample size**	0.0046027	0.0028686	1.60	0.127	− 0.0014494.0106548
**_cons**	0.0992271	0.0620645	1.60	0.128	− 0.0317175.2301718
**Year**	− 0.0345581	0.0362611	-0.95	0.354	− 0.1110622.0419461
**_cons**	70.04256	73.30167	0.96	0.353	-84.61043224.6956

### Publication bias

Publication bias was assessed through subjective examination of the funnel plot (Fig. 6) and Egger’s regression test, revealing a *p*-value of < 0.001, indicative of publication bias. Subsequent trim and fill analysis, incorporating ten additional studies, suggested the existence of missed small studies. This adjustment potentially lowers the prevalence of meropenem-resistant *P. aeruginosa* to 3.7% (95% CI: -2.3, 9.6).

### Sensitivity analysis

Sensitivity analysis was performed on the prevalence of meropenem-resistant *P. aeruginosa* using a random effects model (Table 3). Each of the excluded studies exhibited minor variations in the prevalence of meropenem-resistant *P. aeruginosa* in Ethiopia.

**Table 3:** Sensitivity analysis of studies included for estimation of pooled prevalence of meropenem resistant *P. aeruginosa* in Ethiopia

Study omitted	Estimate	[95% Conf. Interval]	
Gashaw et al [34]	0.14243186	0.0881113	0.19675241
Legesse et al [28]	0.15922157	0.1016699	0.21677324
Motbainor. Et al [30]	0.20910977	0.0773275	0.34089205
Mekonnen et al [29]	0.14995179	0.09382511	0.20607847
Alebel et al [27]	0.14969699	0.09391756	0.20547643
Abdeta et al [40]	0.14658256	0.09069896	0.20246616
Alemayehu. Et al [38]	0.14505221	0.09029172	0.1998127
Mitiku et al [39]	0.14585564	0.09048406	0.2012272
Abda et al [26]	0.15089363	0.09481937	0.20696789
Bizuayehu et al [43]	0.15028805	0.09435938	0.20621672
Abayneh et al [37]	0.17417368	0.11178836	0.236559
Worku et al [33]	0.17417368	0.11178836	0.236559
Tilahun et al [32]	0.1223927	0.07197122	0.17281418
Beshah et al [41]	0.1467315	0.09193183	0.20153117
Tilahun [31]	0.12002805	0.07038046	0.16967565
Sewunet et al [36]	0.1599513	0.10163819	0.21826442
Mussema et al [35]	0.17833784	0.11322875	0.24344693
Mekonnen et al [44]	0.17417368	0.11178836	0.236559
Beshah et al [42]	0.1467315	0.09193183	0.20153117
Combined	**0.15086962**	**0.0961069**	**0.20563234**

### Trim and fill analysis

A trim and fill analysis was carried out to evaluate the influence of overlooked studies on the prevalence of meropenem-resistant *P. aeruginosa* in Ethiopia. The pooled estimates of prevalence were adjusted from 15.1 to 3.7% (Table 4).

**Table 4:** Trim and fill analysis of prevalence of meropenem resistant *P. aeruginosa* in Ethiopia

Method		Pooled Est	95% CI		Asymptotic		No. of Studies
			Lower	Upper	z_value	*p*_value	
**Before**	Fixed	0.029	0.013	0.045	3.515	0.000	19
	Random	0.151	0.096	0.206	5.400	0.000	
**Filled**	Fixed	0.011	-0.005	0.027	1.344	0.179	28
	Random	0.037	-0.023	0.096	1.212	0.226	

Test for heterogeneity: Q = 216.375 on 27 degrees of freedom (*p* = 0.000)

Moment-based estimate of between studies variance = 0.015

## Discussion

The global landscape of public health faces a pressing and alarming threat in the form of antimicrobial resistance, a phenomenon that exacts a toll on mortality and morbidity rates, particularly in low- and middle-income countries where diagnostic access is constrained, and regulation of antimicrobial prescription and intake remains insufficiently established [2]. Within this context, carbapenems emerge as pivotal agents in the treatment of *P. aeruginosa* infections [45]. Our understanding deepens as we recognize that no single carbapenem exhibits superiority over another in preventing the emergence of carbapenem resistance [46].

Furthermore, the ominous implications of meropenem resistance extend to being a significant predictor of in-hospital mortality, coupled with a notable escalation in hospital costs associated with carbapenem resistance [47]. The specter of cross-infection looms within hospital settings, emphasizing the critical importance of implementing stringent infection control measures to mitigate the spread of resistant strains [48].

The findings of our systematic review and meta-analysis on the prevalence of meropenem-resistant *P. aeruginosa* in Ethiopia reveal crucial insights into the landscape of antimicrobial resistance in this context. The overall pooled prevalence of meropenem-resistant *P. aeruginosa* stands at 15%, underscoring the significant burden of resistance within the country.

A comparative perspective is provided by the Meropenem Yearly Susceptibility Test Information Collection (MYSTIC) Program in the United States, which reports a meropenem susceptibility rate of 85.4% against *P. aeruginosa* based on an analysis of 439 strains [49]. This rate aligns closely with the pooled susceptibility rate observed in our study.

However, the global scenario unveils higher prevalence rates of carbapenem resistance against *P. aeruginosa* in diverse regions. In Europe, susceptibility data collected from 29 European Intensive Care Units (ICUs) participating in the MYSTIC Program (1997–2000) indicate favorable activity of meropenem (76.1%) and imipenem (68.2%) against *P. aeruginosa*, albeit with noteworthy inter-country variations [50]. A study in Latin America reveals susceptibility rates of 57% for meropenem and 52% for imipenem among *P. aeruginosa* isolates [51]. Similarly, antimicrobial susceptibility data from China demonstrate susceptibility rates ranging from 71.5 to 80.5% for meropenem and 75.3–77.2% for imipenem in *P. aeruginosa* isolates [52]. In Turkey, additional antimicrobial susceptibility data also indicated a 30% resistance rate of *P. aeruginosa* to meropenem [17], contrasting with a lower reported resistance rate of 8% in sub-Saharan regions of Africa [53].

Notably, our subgroup analysis by the source of infection sheds light on the varying degrees of resistance in different settings. Hospital-acquired infections exhibit a markedly higher prevalence at 28%, emphasizing the heightened risk and challenges associated with nosocomial transmission of meropenem-resistant strains. In contrast, community-acquired infections display a lower but still substantial prevalence of 6%, indicating that resistance is not confined solely to healthcare settings but extends to the broader community.

Our findings suggest that the prevalence of meropenem-resistant *P. aeruginosa* in hospital-acquired infections is significantly elevated at 28% in Ethiopia compared to Africa (13.7%). In the Eastern Mediterranean, the prevalence reaches 32.7%, while, in the South-East Asia, it stands at 38.4%. Notably, the prevalence is even higher in the West-Pacific regions, reaching 60.6% [54].

Further exploration through subgroup analysis based on geographical regions and sample types provides a nuanced understanding of the regional and specimen-specific variations in meropenem resistance. The Amhara region emerges as a hotspot with a prevalence of 23%, highlighting the need for targeted interventions in this area. This regional disparity may be attributed to local factors such as healthcare practices, antimicrobial usage patterns, and infection control measures.

Moreover, the variance in prevalence between different sample types is noteworthy. Wound swabs, as a sample type, exhibit a higher prevalence of 25%, suggesting that wounds may serve as reservoirs for meropenem-resistant *P. aeruginosa*. This finding highlights the importance of vigilant monitoring and effective infection control measures in wound care settings.

Conversely, the Oromia region reports a lower prevalence of 7%, indicating regional heterogeneity in resistance patterns. Similarly, the sputum sample type shows a lower prevalence of 5%, suggesting potential variations in resistance mechanisms across different specimen types.

The observed variations in prevalence across subgroups underscore the complex and multifaceted nature of antimicrobial resistance. The higher prevalence in hospital settings and specific regions necessitates a targeted approach to enhance infection control measures, optimize antibiotic stewardship, and address the contextual factors contributing to resistance. Additionally, the identification of higher resistance in certain sample types calls for heightened surveillance and tailored strategies in clinical practice.

Even though, findings of this systematic review and meta-analysis provides valuable insights into the challenges posed by antimicrobial resistance in Ethiopia the analysis encountered a notable limitation due to the presence of significant heterogeneity among the included studies, as indicated by a high I^2^ value of 83.6% and a *p*-value < 0.001.

In an effort to address this heterogeneity, a weighted inverse variance random-effects model was initially applied. However, despite this attempt, the heterogeneity persisted, prompting further investigation into the potential sources of variability. A forest plot, serving as a subjective assessment tool, was employed to visually inspect the distribution of effect sizes across studies. Additionally, subgroup analysis was conducted to explore whether specific characteristics of the studies contributed to the observed heterogeneity.

In the course of the subgroup analysis, it became evident that certain factors may be influencing the variation in study outcomes. Univariate meta-regression was subsequently employed to systematically evaluate the impact of key variables, including the number of isolates and publication years, on the observed heterogeneity. The results of the meta-regression revealed that the number of isolates significantly contributed to the source of heterogeneity. Differences in sample sizes could potentially lead to fluctuations in resistance rates, contributing to the observed heterogeneity. It is imperative to acknowledge that studies with larger sample sizes may have more robust estimates, while smaller studies may be more susceptible to random variations.

Results of this study also revealed noteworthy findings related to publication bias. The initial evaluation of publication bias, conducted through a subjective assessment of the funnel plot and Egger’s regression test, yielded a *p*-value of < 0.001, indicative of the presence of bias in the included studies. Recognizing the potential impact of such bias on the robustness of the results, a trim and fill analysis was undertaken. This analysis suggested the existence of missed small studies, and upon their inclusion, the recalibrated prevalence of meropenem-resistant *P. aeruginosa* was adjusted to 3.7%.

In addition to publication bias, sensitivity analysis was conducted to assess the robustness of the prevalence estimates by applying a random effects model. Interestingly, the results of the sensitivity analysis indicated that each of the excluded studies did not show a significant difference in the pooled prevalence. This finding suggests that the exclusion of individual studies did not disproportionately influence the overall prevalence estimate, reinforcing the stability of the meta-analytic findings.

### Strength and limitations of the study

This systematic review and meta-analysis stands out for its thoroughness, representing the inaugural discovery on meropenem-resistant *P. aeruginosa* in Ethiopia. This meta-analysis on meropenem-resistant *P. aeruginosa* in Ethiopia offers valuable insights, yet it faces notable limitations that warrant consideration for a nuanced interpretation. A primary concern is the significant heterogeneity among included studies, stemming from diverse methodologies, population characteristics, and study designs. This diversity calls for caution in generalizing the pooled prevalence estimate, recognizing potential biases. Additionally, a notable limitation involves the prevalent use of the disk diffusion method over the recommended minimum inhibitory concentration (MIC) method in primary studies. This methodological difference introduces variability and may affect result accuracy and comparability across studies. Acknowledging these limitations is essential for a comprehensive grasp of our meta-analysis findings. Despite challenges, our study serves as a basis for future research, emphasizing the importance of standardized methodologies and increased adherence to recommended testing techniques.

## Conclusion

Our comprehensive meta-analysis on the prevalence of meropenem resistance in *P. aeruginosa* within the Ethiopian context reveals important insights. The pooled prevalence of meropenem resistance across the country is determined to be 15%. Subgroup analysis further elucidates regional and infection context disparities, indicating potential differences in resistance patterns based on geography and infection acquisition settings. Overall, our meta-analysis provides a comprehensive overview of the current landscape of meropenem resistance *P. aeruginosa* in Ethiopia. These findings serve as a foundation for targeted interventions, regional policies, and further research aimed at addressing and mitigating the challenges posed by antimicrobial resistance in the country.

## Data Availability

No datasets were generated or analysed during the current study.
